# The Construction and Application of a Clinical Decision Support System for Cardiovascular Diseases: Multimodal Data-Driven Development and Validation Study

**DOI:** 10.2196/63186

**Published:** 2025-03-03

**Authors:** Shumei Miao, Pei Ji, Yongqian Zhu, Haoyu Meng, Mang Jing, Rongrong Sheng, Xiaoliang Zhang, Hailong Ding, Jianjun Guo, Wen Gao, Guanyu Yang, Yun Liu

**Affiliations:** 1 School of Computer Science and Engineering, Southeast University, No.2 Sipailou, Nanjing, 210096, China, 86 02552090872; 2 Department of Information, The First Affiliated Hospital of Nanjing Medical University, Nanjing, China; 3 Department of Quality Management, The First Affiliated Hospital of Nanjing Medical University, Nanjing, China; 4 Department of Cardiology, The First Affiliated Hospital of Nanjing Medical University, Nanjing, China; 5 Department of Oncology, The First Affiliated Hospital of Nanjing Medical University, Nanjing, China; 6 Department of Geriatrics Endocrinology, The First Affiliated Hospital of Nanjing Medical University, Nanjing, China

**Keywords:** CVD, CDSS, multimodel data, knowledge engine, development, cardiovascular disease, clinical decision support system

## Abstract

**Background:**

Due to the acceleration of the aging population and the prevalence of unhealthy lifestyles, the incidence of cardiovascular diseases (CVDs) in China continues to grow. However, due to the uneven distribution of medical resources across regions and significant disparities in diagnostic and treatment levels, the diagnosis and management of CVDs face considerable challenges.

**Objective:**

The purpose of this study is to build a cardiovascular diagnosis and treatment knowledge base by using new technology, form an auxiliary decision support system, and integrate it into the doctor’s workstation, to improve the assessment rate and treatment standardization rate. This study offers new ideas for the prevention and management of CVDs.

**Methods:**

This study designed a clinical decision support system (CDSS) with data, learning, knowledge, and application layers. It integrates multimodal data from hospital laboratory information systems, hospital information systems, electronic medical records, electrocardiography, nursing, and other systems to build a knowledge model. The unstructured data were segmented using natural language processing technology, and medical entity words and entity combination relationships were extracted using IDCNN (iterated dilated convolutional neural network) and TextCNN (text convolutional neural network). The CDSS refers to global CVD assessment indicators to design quality control strategies and an intelligent treatment plan recommendation engine map, establishing a big data analysis platform to achieve multidimensional, visualized data statistics for management decision support.

**Results:**

The CDSS system is embedded and interfaced with the physician workstation, triggering in real-time during the clinical diagnosis and treatment process. It establishes a 3-tier assessment control through pop-up windows and screen domination operations. Based on the intelligent diagnostic and treatment reminders of the CDSS, patients are given intervention treatments. The important risk assessment and diagnosis rate indicators significantly improved after the system came into use, and gradually increased within 2 years. The indicators of mandatory control, directly became 100% after the CDSS was online. The CDSS enhanced the standardization of clinical diagnosis and treatment.

**Conclusions:**

This study establishes a specialized knowledge base for CVDs, combined with clinical multimodal information, to intelligently assess and stratify cardiovascular patients. It automatically recommends intervention treatments based on assessments and clinical characterizations, proving to be an effective exploration of using a CDSS to build a disease-specific intelligent system.

## Introduction

The high incidence of cardiovascular disease (CVD) is an important public health problem worldwide. CVD remains the leading cause of mortality worldwide and a major contributor to disability. CVD was responsible for 18.6 million deaths according to the 2019 Global Burden of Disease study. China is one of the countries most burdened by CVD [1]. In 2021, approximately 5.1 million individuals lost their lives to CVD in China [2]. Thus, it is of great significance to raise awareness of CVD and screening individuals at risk for CVD so that effective interventions and strategies can be implemented. CVD is influenced by risk factors such as tobacco use, an unhealthy diet, physical inactivity, obesity, elevated blood pressure, abnormal blood lipids, and elevated blood glucose [3,4]. In 2007, the World Health Organization published guidelines for the assessment and management of cardiovascular risk that provide guidance for reducing disability and premature deaths from CVD in people at high risk who have not yet experienced a cardiovascular event [5]. In addition, new artificial intelligence technology for CVD prediction and risk management has been attempted [6,7].

The 2022 China Cardiovascular Health and Disease Report Summary highlights that the rapid aging of the population and the widespread prevalence of unhealthy lifestyles have resulted in a significant portion of the population being at risk for CVD, and the burden of CVD continues to rise, as evidenced by persistently high prevalence rates, increasing mortality, and escalating treatment and management costs [8-10]. The prevention and treatment of CVDs in China presents significant challenges. In recent years, several medical policies have prioritized this issue. From 2021 to 2023, the China National Health Commission made “improving the reperfusion treatment rate for acute ST-segment elevation myocardial infarction (MI)”, “improving the reperfusion treatment rate for acute ischemic stroke”, and “improving the standardized prevention rate for venous thromboembolism (VTE)” in the national top ten medical quality and safety improvement objectives. Additionally, the “Opinions on Promoting High-Quality Development of Public Hospitals” issued by the State Council Office in May 2021, emphasized cardiovascular and cerebrovascular diseases as key clinical specialties for the high-quality development of public hospitals.

Clinical decision support systems (CDSS) leverage health information technology to provide health care professionals with intelligent decision-making tools, aiming to improve medical quality, reduce errors, and improve patient outcomes [11-13]. CDSS can offer various forms of support, including diagnostic recommendations, treatment plan selection, and medication dosage calculations, based on a comprehensive medical knowledge database and patient-specific data. The primary objective of CDSS is to optimize clinical decisions by integrating clinical observations with knowledge bases, ultimately improving patient outcomes [14]. In recent years, CDSS has been preliminarily applied in some specific medical scenarios, such as chronic obstructive pulmonary disease patient management, antibiotic management, and venous thromboembolism management [15-17]. In the process of prevention, diagnosis, and treatment of CVDs, some researchers have also used Machine Learning, big data and other new technologies to carry out research on the prediction of Major Adverse Cardiovascular Events and disease onset [18-20].

Currently, there are several challenges in the diagnosis, treatment, and management of CVD. Uneven allocation of medical resources in different regions leads to large gaps in diagnosis and treatment levels. Insufficient disease awareness leads to a low rate of early CVD diagnosis, while reliance on manual risk assessment forms leads to inefficient risk prediction. The standardization of clinical diagnosis and treatment requires improvement. The quality control method relies on sampling and examination of terminal medical records, which is backward and passive. Therefore, this study proposes the use of CDSS technology combined with multimodal clinical information to intelligently assess and stratify cardiovascular patients. By integrating CDSS with the hospital’s doctor workstation system, auxiliary diagnosis, and treatment are seamlessly incorporated into clinical workflows, leveraging a powerful knowledge base and intelligent models to provide physicians with intelligent recommendations. Based on these assessments and clinical characteristics, it intelligently recommends intervention treatments, offering novel insights for prevention, treatment, and management strategies for CVDs.

## Methods

### CDSS Design

**Figure 1.**
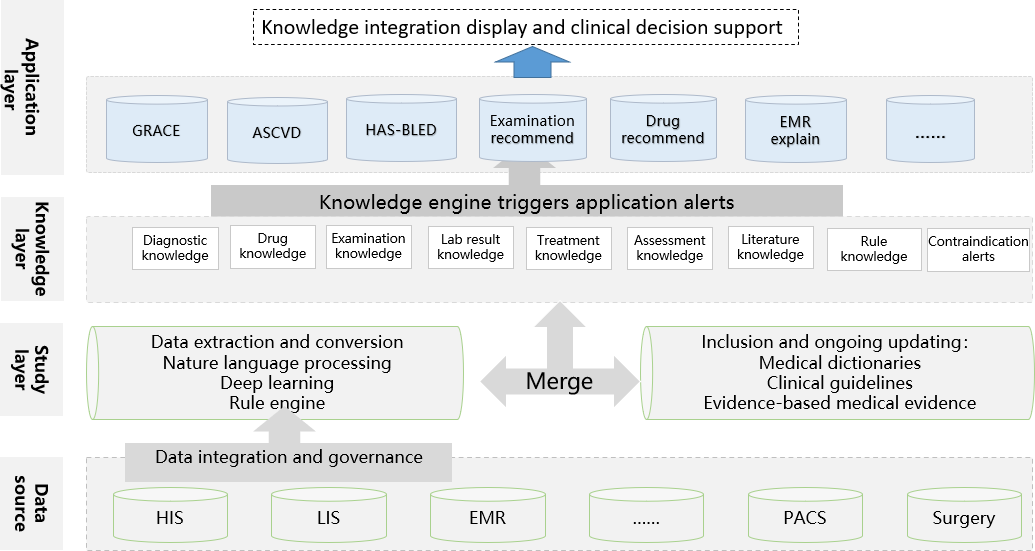
The design of clinical decision support system for cardiovascular disease. ASCVD: atherosclerotic cardiovascular disease; EMR: electronic medical record; GRACE: Global Registry of Acute Coronary Events; HAS-BLED: hypertension, abnormal renal or liver function, stroke, bleeding history or predisposition, labile international normalized ratio, elderly (>65 years), drugs or alcohol concomitantly; HIS: hospital information system; LIS: laboratory information system; PACS: picture archiving and communication system.

The CDSS in the study has a 4-tier architecture shown in Figure 1. Initially, the foundation of decision support is rich data information at the data source layer. Information such as medical orders, medical records, lab tests, examinations, surgery records, etc, are collected and integrated through hospital information integration platforms and data centers. For historical data, the Extract-Transform-Load tool was used to integrate various information systems. For the real-time human-computer interaction data, the data transmit interface was triggered by clicking the temporary save button or mouse movement. For the study layer, based on deep learning, knowledge graphs, rule engines, clinical pathways, and guidelines, the processing process of word segmentation, named entity recognition, and relationship extraction was completed to realize the output of structured variables and data standardization. A triple data set of entity-relationship-entity was formed to construct the clinical knowledge map. Then for the knowledge layer, different types of knowledge representations were established through the previous learning, such as diagnostic knowledge, drug knowledge, examination knowledge, assessment knowledge, literature databases, contraindication alerts, and others. We developed flexible knowledge maintenance tools to maintain the knowledge base according to the characteristics of clinical specialties in hospitals, and provide intelligent recommendation, intelligent early warning, and intelligent audit functions for doctors. Finally, at the application layer, Through the front frontpage parameter docking and the message transmission of the data integration platform, the doctor workstation information is encrypted and transmitted to the CDSS, and the CDSS intelligent prompt module is triggered when ordering medical orders, writing medical records, transferring departments, operating and discharging for each patient of the doctor system. Intelligent decision support is triggered like drug recommendations, examination recommendations, Global Registry of Acute Coronary Events (GRACE) evaluation, atherosclerotic cardiovascular disease (ASCVD) evaluation, etc.

The system is specifically designed for patients who are hospitalized due to CVDs, and it can facilitate diagnosis and treatment by incorporating their comprehensive medical data.

### Ethical Considerations

The study was approved by the Ethics Committee of the First Affiliated Hospital of Nanjing Medical University (2023-SR-743). As this study did not involve a process in which informed consent must be obtained for routine care and the contents of the study do not involve personal privacy, the ethics committee waived the requirement for written informed consent.

### Multimodal Data Fusion Processing

**Figure 2.**
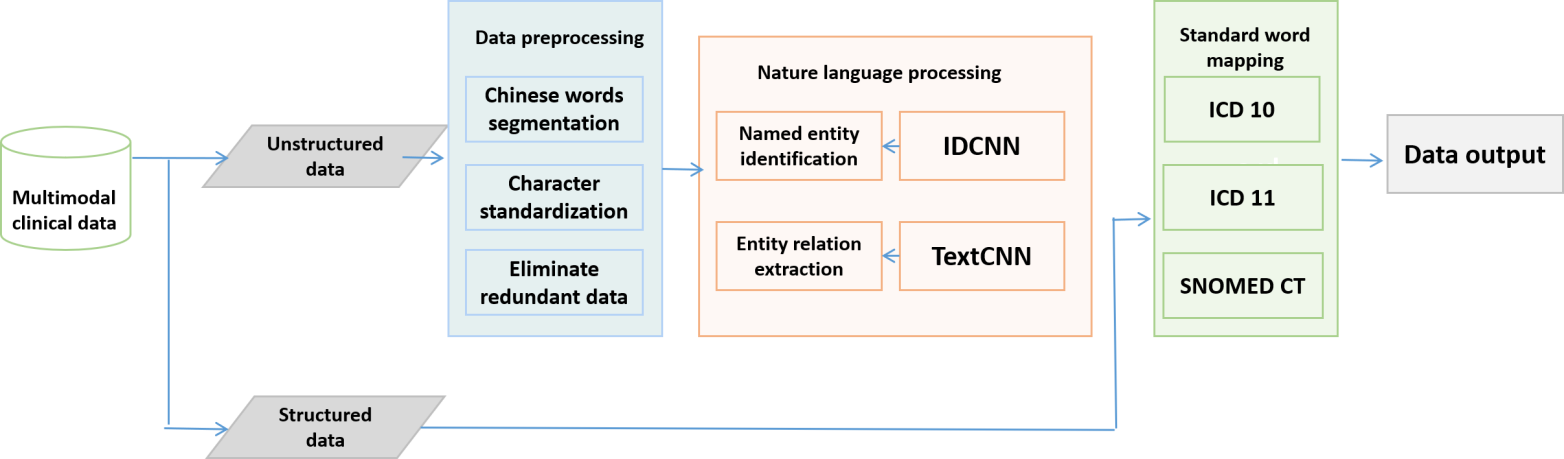
The natural language processing process of multimodal clinical data. ICD: International Classification of Diseases; IDCNN: iterated dilated convolutional neural network; SNOMED CT: Systematized Nomenclature of Medicine – Clinical Terms; TextCNN: text convolutional neural network.

Artificial intelligence (AI) technology is used to integrate multimodal data related to CVD diagnosis- which is dispersed across various hospital systems including hospital information systems, electronic medical records (EMR), laboratory information systems, PACS (picture archiving and communication system), and nursing systems. Through natural language processing (NLP) and medical terminology mapping, a knowledge model is developed. The structure of the NLP module, core deep learning algorithms, and resources are presented in Figure 2. First, preprocessing is performed on unstructured text, including sentence segmentation for word segmentation, standardization of symbols, uniformization of capitalization, reorganization of standardized vocabulary, elimination of less meaningful parts, and formation of summaries that express the main meaning. Then, the named entity recognition model using the BiLSTM-CRF (bidirectional long short-term memory - conditional random field) and IDCNN (iterated dilated convolutional neural network) architecture to extract medical entity words such as time, anatomical site, diagnosis, drug name, drug dose, and surgical name [21]. Next, the entity relationship linking model uses the TextCNN (text convolutional neural network) architecture to extract medical entity combination relationships such as diagnosis-anatomical site-position-time as a group [22]. Then, the core entities such as diagnosis, laboratory tests, drug names, and attribute entity words such as time, existence status, and drug dosage are mapped to medical standard words. Finally, according to entities and the relationship between entities, a disease auxiliary decision support model using CNN-DSSM (convolutional neural network - deep semantic similarity model) is constructed, which can provide a list of diagnostic probabilities and recommend reasonable examinations according to the patient’s chief complaints and medical history, to assist doctors in diagnosis and reduce the probability of missed diagnosis and misdiagnosis.

### CVD Assessment Models and Recommendations

This study focuses on the diagnosis and treatment of common CVDs by incorporating assessment models such as GRACE, ASCVD, CHA2DS2-VASc (congestive heart failure, hypertension, age ≥75 years [doubled], diabetes, stroke [doubled], vascular disease, age 65 to 74 years and sex category [female]) for stroke risk evaluation in patients with nonvalvular atrial fibrillation, alongside other intelligent diagnostic and treatment recommendation systems [23-25]. Using the stratified GRACE risk assessment as an example [26-28], NLP technology was applied to gather data from various clinical systems, including age, heart rate, systolic blood pressure, serum creatinine, positive initial heart markers, ST segment changes, cardiac arrest at admission and Killip class [29,30]. Based on the risk assessment outcomes, distinct levels of low risk, medium risk, high risk, and extremely high risk were established. Subsequently, as shown in Table 1, corresponding quality control strategies and treatment plan recommendations were developed for each risk category.

**Table 1.** GRACE (Global Registry of Acute Coronary Events) risk assessment results and related recommendations.

Risk level	GRACE score	In-hospital mortality (%)
Low risk	≤108	<1
Moderate risk	109‐140, or combined with diabetes mellitus, renal insufficiency, LVEF^a^ <40%, chronic cardiac insufficiency, angina pectoris after early myocardial infarction (MI), PCI^b^ history, or CABG^c^ history	1‐3
High risk	>140, or the presence of MI-related troponin rise or fall, ST-T^d^ dynamic changes (with or without symptoms)	>3
Extremely high risk	Regardless of score, Presence of any of the following: hemodynamic instability or cardiogenic shock, recurrent or persistent chest pain refractory to medical therapy, fatal arrhythmia or cardiac arrest, MI with mechanical complications, acute heart failure, recurrent ST-T dynamic changes (especially intermittent ST-segment elevation)	>3

a LVEF: left ventricular ejection fraction.

b PCI: percutaneous coronary intervention.

c CABG: coronary artery bypass grafting.

d ST-T: electrocardiogram ST-T wave.

### Rule Inference Engine

The study uses a clinical decision knowledge engine framework to facilitate the reasoning and computation of clinical rules in the form of decision trees. Rule engine is a branch of an expert system, that is developed from an inference engine. It solves practical problems by simulating human thinking mode and expresses the results of inference in an understandable form. IBM Operational Decision Manager is used as a tool prototype to input, manage, and maintain the business rules of the system users, including rule modeling, writing, testing, deployment, and maintenance, and provides rule base mechanism for the organization, storage, and execution of business rules [31]. Through Operational Decision Manager, clinical knowledge rules are modeled, developed, and validated. After data collection and cleaning, a standardized data archive is realized. The rule inference engine makes logical operations based on the transformed standard term set. The output reminders were used to assist clinicians in their diagnosis and treatment decisions.

### Application Assessment

Before the CDSS was officially launched, an independent panel of senior experts was invited to assess whether the recommendations were consistent with the current medical standards, practice guidelines, and best clinical practices. The experts could provide their own professional opinions and suggestions for improvement. After a comprehensive evaluation of expert opinions, the decision should be made whether to update and optimize the existing knowledge rules. After the system’s official launch, we conducted a comparative analysis between CDSS recommendations and doctors’ revised treatment plans. If there is an unreasonable knowledge base, after review by the quality control group, it was revised to improve the knowledge base for better decision support.

In terms of quality monitoring for daily use, a big data analytic platform is established and multidimensional statistic results are used to support management decision-making. The platform analyzes various quality control indicators, including the dynamic assessment rate of risk stratification. Hospital managers can review the control of CVD risk and the quality of diagnosis and treatment within the institution from multiple dimensions such as departments, doctors, types of diseases, and results of risk assessments, to formulate targeted management plans.

### Statistical Analyses

The key performance indicators are presented as numbers (%) for categorical data. We compared the key performance indicators using Cochran-Armitage trend test. The threshold for statistical significance was *P*<.05. All statistical tests were performed using R (version 4.4.2; R Foundation).

## Results

### Real-Time Intelligent Triggering

**Figure 3.**
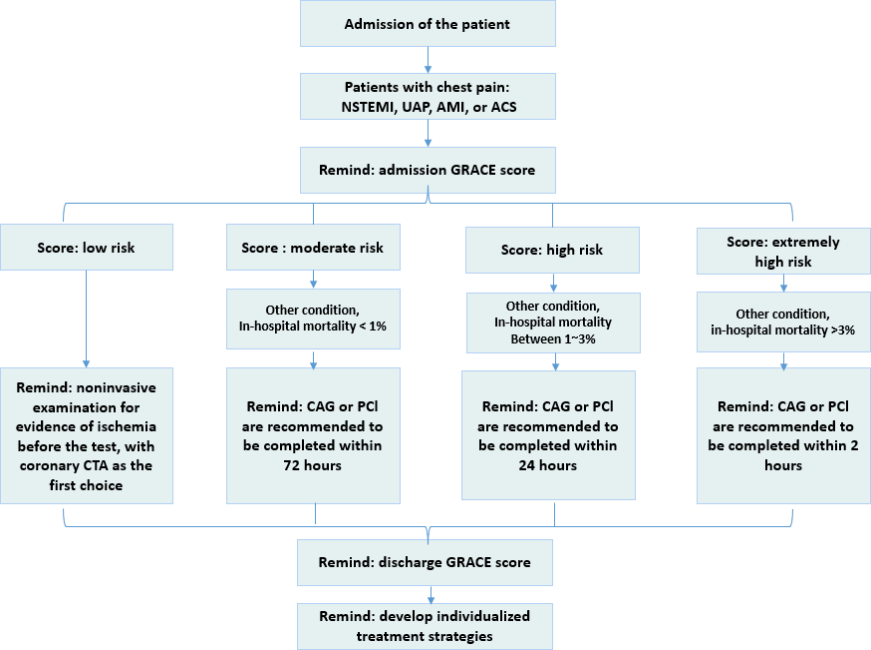
A real-time intelligent triggering sample knowledge path. ACS: acute coronary syndrome; AMI: acute myocardial infarction; CAG: coronary angiogram; CTA: computed tomography angiography; GRACE: Global Registry of Acute Coronary Events; NSTEMI: non–ST-elevation myocardial infarction; PCI: percutaneous coronary intervention; UAP: unstable angina pectoris.

The CDSS is integrated into the clinical workflow, linking key processes such as admission, operation, transfer, discharge, and other clinical activities. It enables real-time data flow integration and message triggering within the interfaces for prescribing and medical record documentation. During the assessment phase, when the system identifies a patient with a specified diagnosis, the CDSS triggers evaluation tools such as GRACE, ASCVD, and CHA2DS2-VASc. It performs a preassessment based on the patient’s medical history and provides traceable evidence from original medical records, prompting the doctor to verify the results. If the patient’s CVD risk stratification outcome changes, the system alerts the doctor to reassess the patient. In the diagnosis and treatment phase, based on the patient’s current assessment and clinical status, it recommends appropriate diagnostic and treatment measures. Figure 3 illustrates an example of real-time intelligent knowledge path triggering.

### Three-Level Control Management

To achieve precise quality control for different risk levels, our study proposed a 3-level control management system for in-process alerts. The 3-level management approach involves establishing distinct levels of control for different regulations based on the level of risk or degree of emergency and providing varying degrees of reminders to medical professionals. The specifics are as follows: Level 1 control triggers a small red dot on the EMR interface, signaling the doctor’s attention and requiring active selection. Level 2 control involves an active pop-up window on the EMR interface that the doctor can clearly see and address with the option to close and continue other tasks. Level 3 control features an active pop-up window that the doctor must address or provide a reason for dismissing before proceeding with other tasks. The 3-level control management issues alerts based on varying risk levels and incorporates the timing requirements for doctor’s medical record documentation. This system prompts the doctor to complete the relevant assessment, diagnosis, and treatment actions within the specified time range of the evaluation node.

### Application Effects

#### Overview

The multimodal data-driven accurate CDSS for CVDs was officially launched around the year 2022, while different modules were launched at different specific times. Table 2 lists a before-and-after comparison table of key performance indicators. The important evaluation indicators in the diagnosis and treatment of CVDs such as rate of GRACE assessment (*P*<.001), rate of ASCVD risk stratification assessment (*P*<.001), rate of interventional therapy within 2 hours after acute MI (*P*<.001), rate of lipid-lowering drugs treatment for coronary heart disease (*P*<.001), rate of blood pressure well-control in hypertensive patients (*P*<.05), rate of interventional therapy within 2 hours after acute MI (*P*<.001), etc were significantly improved after the system came into use. The evaluation indicators after 2 years were better than those after 1 year. The indicators under 3-level mandatory control, such as the rate of CHA2DS2-VASc stroke risk assessment and rate of HAS-BLED (hypertension, abnormal renal or liver function, stroke, bleeding history or predisposition, labile international normalized ratio, elderly [>65 years], drugs or alcohol concomitantly) bleeding risk assessment, were directly becoming 100% after CDSS was online.

**Table 2.** The before-and-after comparison table of key performance indicators.

Name of indicators	Before CDSS^a^ was online, n/N (%)	After CDSS was online (1 year), n/N (%)	After CDSS was online (2 year), n/N (%)	*χ*² (*df*)	*P* value
Rate of GRACE^b^ assessment at admission	290/426 (68)	386/429 (90)	485/506 (96)	138.22 (2)	<.001
Rate of GRACE assessment at discharge	304/426 (71)	386/429 (90)	501/506 (99)	159.11 (2)	<.001
Rate of coronary CTA^c^ in MI^d^ patients	37/166 (22)	106/200 (53)	198/246 (81)	136.82 (2)	<.001
Rate of interventional therapy within 2 h after atecute MI	35/57 (61)	54/65 (83)	57/60 (95)	25.884 (2)	<.001
Rate of ASCVD^e^ risk stratification assessment	588/937 (63)	745/780 (96)	1058/1100 (96)	422.64 (2)	<.001
Rate of blood pressure well-control in hypertensive patients	162/213 (76)	269/336 (80)	385/460 (84)	5.71 (2)	<.05
Rate of lipid-lowering drugs treatment for coronary heart disease	347/673 (52)	500/733 (68)	676/821 (82)	161.71 (2)	<.001
Rate of CHA2DS2-VASc^f^ stroke risk assessment	83/88 (94)	210/210 (100)	222/222 (100)	15.08 (2)	<.001
Rate of HAS-BLED^g^ bleeding risk assessment	—^h^	177/177 (100)	166/166 (100)	—	—
Rate of diagnosis and early warning diagnosis of atrial fibrillation	—	147/158 (93)	296/313 (94)	—	—
Rate of stroke prevention for atrial fibrillation (medication or NOAC^i^) (high bleeding risk)	33/44 (75)	57/68 (84)	41/50 (82)	0.68 (2)	.41
Rate of stroke prevention in atrial fibrillation (medication or NOAC) (low bleeding risk)	79/96 (82)	81/95 (85)	162/174 (93%)	7.64 (2)	<.01

a CDSS: clinical decision support system.

b GRACE: Global Registry of Acute Coronary Events.

c CTA: computed tomography angiography.

d MI: myocardial infarction.

e ASCVD: atherosclerotic cardiovascular disease.

f CHA2DS2-VASc: congestive heart failure, hypertension, age ≥75 years (doubled), diabetes, stroke (doubled), vascular disease, age 65 to 74 years, and sex category (female).

g HAS-BLED: hypertension, abnormal renal or liver function, stroke, bleeding history or predisposition, labile international normalized ratio, elderly (>65 years), drugs or alcohol concomitantly.

h Not available.

i NOAC: nonvitamin K oral anticoagulants.

The CDSS function in our hospital has produced the following outcomes.

#### Timely Evaluation

Complete a GRACE score assessment for chest pain patients considered for non-ST segment elevation acute coronary syndrome, to evaluate the comprehensive risk of all-cause mortality or the combined risk of all-cause death and MI. For other patients, the system assesses the in-hospital mortality risk within 24 hours of admission and re-evaluates the risk of death within 1 week before discharge and 6 months after discharge.

#### Blood Lipid Screening for High-Risk Patients

Following completing the CVD risk stratification assessment, the system prompts the doctor to perform a blood lipid test. For patients with abnormal blood lipid test data, the system alerts the doctor to diagnose the patient with “dyslipidemia.”

#### Blood Glucose Control Reminder

After reviewing the patient’s hemoglobin and insulin levels, the system prompts the doctor to carry out measures for blood glucose control. The reminder will disappear automatically after the patient’s blood glucose index has reached the target value.

#### Reminder of Antihypertensive Treatment

If the patient is diagnosed with hypertension, the system prompts the doctor to carry out antihypertensive treatment, and the remainder will disappear automatically when the patient’s blood pressure reaches the target value.

#### Timely Feedback Optimization

The system incorporates a user feedback function, enabling doctors to submit web-based feedback when encountering issues or providing suggestions during use. This facilitates the formation of diverse teams for problem-specific optimization and allows users to articulate the benefits derived from using the system. Feedback is searched, analyzed, and responded to based on various criteria such as time of submission, department affiliation, feedback provider, product function, and treatment type in order to ensure continuous improvement.

## Discussion

### The Benefits of CDSS

The implementation of CDSS has significantly enhanced the intelligent assessment and risk stratification of CVDs, effectively intervening and alerting in the subsequent diagnosis and treatment of patients.

On one hand, it has improved the efficiency of CVD risk assessment. Previously, this process required manual completion of scoring forms, which resulted in low efficiency. The AI-based CDSS, however, can analyze medical records and automatically complete the assessment in real-time, greatly enhancing both the efficiency and quality of CVD risk assessment. It has increased the diagnosis rate of CVD by deploying the CDSS across outpatient, emergency, and inpatient physician workstations, and improves the problem of low early diagnosis rate of CVD due to insufficient awareness of non-CVDs among noncardiology doctors. On the other hand, it enhances the standardization of clinical diagnosis and treatment. By identifying risks, issuing real-time warnings, and providing treatment recommendations, it effectively guides doctors in delivering precise treatment according to risks and clinical guidelines, and improves doctors’ compliance with guidelines and standardization of diagnosis and treatment. Regarding quality control, traditional methods relied on sampling terminal medical records were passive and outdated. In contrast, the CDSS big data analysis platform can view the risk control and treatment quality of CVDs from the dimensions of departments, doctors, diseases, and risk assessment results, and allows quality control effect in real time.

Through automatic collection and real-time evaluation of key medical data in the process of diagnosis and treatment, the system establishes a new model of CVD quality control with information sharing and hierarchical treatment. It can be predicted that the application of information tools such as CDSS can help establish standard data sets for CVDs, support medical institutions to establish real-world clinical research platforms for CVDs, and aid specialized centers in conducting high-quality clinical research.

### The Comparisons With Related Work

We have compared the characteristics of cardiovascular-related CDSS in recent years in Table 3. The innovation of the systems in our study lies in hierarchical evaluation and multistep reminders. We integrated multimodal data from the hospital laboratory information system, hospital information system, EMR, electrocardiography, nursing, and other systems to build the knowledge model. We established a 3-level control management system for in-process alerts as part of the workflow and providing recommendations at critical points of decision-making. The CDSS is integrated into the doctor’s workstation and serves as a supplement or reminder to recommend examinations, tests, treatments, etc during the diagnosis and treatment process. The knowledge base and AI model are constructed from various clinical guidelines and historical patient data; however, personalization of patients may result in data bias, algorithm bias, or cognitive bias during model building. We conducted a comparative analysis between CDSS recommendations and doctors’ revised treatment plans and improved the knowledge base for better decision support.

**Table 3.** The comparisons of cardiovascular-related clinical decision support system (CDSS) in recent years and our study.

First author, year and country of study	Clinical setting	Clinical condition	CDSS type	Study design	Additional description of CDSS
Sperl-Hillen et al (2018), United States [32]	Primary care clinics	Reduce cardiovascular (CV) risk	A web-based, point-of-care CDSS system, based on evidence-based national clinical guidelines seamlessly integrated within the electronic health record (EHR) and primary care workflow	Multicenter, randomized trial	For CDS clinic patients identified algorithmically with high CV risk, rooming staff was prompted by the EHR to print CDS that identified evidence-based treatment options for lipid, blood pressure, weight, tobacco, or aspirin management and prioritized them based on the potential benefit to the patient.
Kharbanda et al (2018), United States [33]	Primary care clinics	Improve the recognition and management of hypertension in adolescents.	Provides CV risk assessments based on EHR data and prompts providers to discuss risk reduction with patients	Multicenter, randomized trial	The recognition of hypertension was identified by an automated review of diagnoses and problem lists and a manual review of clinical notes, antihypertensive medication prescriptions, and diagnostic testing.
Adusumalli et al (2021), United States [34]	Primary care practices	Evaluate the effect of passive choice and active choice interventions in the EHR to promote guideline-directed statin therapy.	Passive and active choice EHR alerts to cardiologists	Multicenter, randomized trial	In passive choice, cardiologists had to manually access an alert embedded in the EHR to select options to initiate or increase statin therapy. In active choice, an interruptive EHR alert prompted the cardiologist to accept or decline guideline-directed statin therapy. Cardiologists in the control group were informed of the trial but received no other interventions.
Ventura et al (2022), Portugal [35]	2 cardiology units	Reduce recurrent cardiovascular events	Online CDSS for remote patient monitoring	Multicenter, randomized trial	This study protocol aims to assess the effectiveness of a user-friendly, comprehensive CDSS for remote patient monitoring of CVD^a^ patients, primarily on the reduction of recurrent cardiovascular events.
Weaver et al (2024), United States [36]	Outpatient oncology	Promote provider-patient CV health (CVH) discussions in outpatient oncology.	Automated heart-health assessment (AH-HA) clinical decision support tool which displayed American Heart Association Life’s Simple 7 CVH factors (BMI, physical activity, diet, smoking status, blood pressure, cholesterol, and glucose), populated from the EHR	Single center, observational	The tool displayed American Heart Association Life’s Simple 7 CVH factors (BMI, physical activity, diet, smoking status, blood pressure, cholesterol, and glucose), populated from the EHR, alongside cancer treatments received with cardiotoxic potential.
This study	Hospital	Prevention and management of cardiovascular diseases	3-level assessment card control for patient admission and predischarge reminders, pop-up windows, and screen domination operations	Single center, observational	This study designed a CDSS system with data, learning, knowledge, and application layers. It integrates multimodal data from hospital LIS (laboratory information system), HIS (hospital information system), EMR (electronic medical records), electrocardiography, nursing, and other systems to build a knowledge model.

a CVD: cardiovascular disease.

### Limitations

Although the CDSS has been applied well in the First Affiliated Hospital of Nanjing Medical University, it still needs to be promoted in more medical and health institutions to verify its effectiveness. Until then, uniform data collection standards and procedures need to be developed to ensure that data, including patient information, treatment regimens, and clinical outcomes, can be collected in a consistent manner across all study sites. In addition, ethics committee approval at each participating institution was required to ensure compliance with local laws and regulations and to protect patient privacy. According to the results of multicenter, the performance of CDSS in different regions and different populations was evaluated, and the possible limitations or improvement space were identified, so as to provide more accurate auxiliary decision support. Tailoring the system to fit the specific workflows and practices of the new setting can improve acceptance and use rates, thereby increasing the likelihood of positive outcomes.

### Conclusions

In this study, AI was used to perform NLP on the clinical medical records of patients, and a cardiovascular specialist CDSS was constructed to automatically identify patients at risk of CVD and improve the diagnostic rate of CVD patients by cardiologists and noncardiologists. It is an effective exploration to build an intelligent system for specialized diseases through CDSS to realize automatic assessment of CVD risk stratification, assist doctors in carrying out corresponding risk assessment and treatment, intervene in doctors’ nonstandard diagnosis and treatment behavior in real time, improve the rate of appropriate lipid-lowering, blood pressure lowering and blood glucose control treatment, improve the prognosis of patients, and provide objective data for hospital management and evaluation of medical quality.

In the next step, when carrying out the construction of specialized precision CDSS, we will continue to improve the knowledge of specialized diseases by in-depth understanding of the diagnosis and treatment characteristics of clinical specialty fields and using the self-defined modules of the medical knowledge base in the CDSS. We will formulate standardized data collection requirements, carry out data transformation mapping, and continue to verify its effectiveness in more medical and health institutions to improve the versatility of the system. This enables refined management of knowledge and enables CDSS to better meet the management and research needs of various specialized diseases.

## Abbreviations

AI: artificial intelligence
ASCVD: atherosclerotic cardiovascular disease
BiLSTM-CRF: bidirectional long short-term memory - conditional random field
CDSS: clinical decision support system
CHA2DS2-VASc: congestive heart failure, hypertension, age ≥75 years (doubled), diabetes, stroke (doubled), vascular disease, age 65 to 74 years, and sex category (female)
CNN-DSSM: convolutional neural network - deep semantic similarity model
CVD: cardiovascular disease
EMR: electronic medical record
GRACE: Global Registry of Acute Coronary Events
HAS-BLED: hypertension, abnormal renal or liver function, stroke, bleeding history or predisposition, labile international normalized ratio, elderly (>65 years), drugs or alcohol concomitantly
IDCNN: iterated dilated convolutional neural network
MI: myocardial infarction
NLP: natural language processing
